# Bioenergy sorghum nodal root bud development: morphometric, transcriptomic and gene regulatory network analysis

**DOI:** 10.3389/fpls.2024.1456627

**Published:** 2024-10-21

**Authors:** Austin Lamb, Evan Kurtz, Priscilla Glenn, Brian A. McKinley, John Mullet

**Affiliations:** Department of Biochemistry and Biophysics, Texas A&M University, College Station, TX, United States

**Keywords:** bioenergy sorghum, nodal root buds, development, transcriptome, gene regulatory network

## Abstract

Bioenergy sorghum’s large and deep nodal root system and associated microbiome enables uptake of water and nutrients from and deposition of soil organic carbon into soil profiles, key contributors to the crop’s resilience and sustainability. The goal of this study was to increase our understanding of bioenergy sorghum nodal root bud development. Sorghum nodal root bud initiation was first observed on the stem node of the 7^th^ phytomer below the shoot apex. Buds were initiated near the upper end of the stem node pulvinus on the side of the stem opposite the tiller bud, then additional buds were added over the next 6-8 days forming a ring of 10-15 nascent nodal root buds around the stem. Later in plant development, a second ring of nodal root buds began forming on the 17^th^ stem node immediately above the first ring of buds. Overall, nodal root bud development can take ~40 days from initiation to onset of nodal root outgrowth. Nodal root buds were initiated in close association with vascular bundles in the rind of the pulvinus. Stem tissue forming nascent nodal root buds expressed sorghum homologs of genes associated with root initiation (*WOX4*), auxin transport (*LAX2, PIN4*), meristem activation (*NGAL2*), and genes involved in cell proliferation. Expression of *WOX11* and *WOX5*, genes involved in root stem niche formation, increased early in nodal root bud development followed by genes encoding PLTs, LBDs (LBD29), LRP1, SMB, RGF1 and root cap LEAs later in development. A nodal root bud gene regulatory network module expressed during nodal root bud initiation predicted connections linking *PFA5*, *SPL9* and *WOX4* to genes involved in hormone signaling, meristem activation, and cell proliferation. A network module expressed later in development predicted connections among *SOMBRERO*, a gene involved in root cap formation, and *GATA19*, *BBM*, *LBD29* and *RITF1*/RGF1 signaling. Overall, this study provides a detailed description of bioenergy sorghum nodal root bud development and transcriptome information useful for understanding the regulation of sorghum nodal root bud formation and development.

## Introduction

Atmospheric carbon dioxide levels have reached their highest level in the past ~800,000 years due in part to emissions associated with the use of fossil fuels (Lindsey, 2019). The Intergovernmental Panel on Climate Change (IPCC) estimated that between 2007 and 2016 agriculture, forestry, and other land-use impacts accounted for ~23% of total net anthropogenic emission of greenhouse gases (GHG) (Arneth et al., 2019). Soils, the largest terrestrial carbon sink, store ~2400 PgC globally (Batjes, 1996), however, in areas of intense cropping such as in the mid-west United States, an estimated ~50% of soil organic carbon (SOC) has been lost in the past 100 years (Sanderman et al., 2017). SOC contributes to favorable soil structure, water and nutrient retention, aeration, soil stability, and plant productivity (Hudson, 1994; Davies, 2017). Therefore, restoring SOC levels of annual cropland soils could enhance agricultural productivity and reduce the rate of increase in atmospheric CO_2_ levels. The accumulation of SOC in the soil profile is dependent on plant root systems, therefore, optimizing root production, distribution in soil profiles, and root-microbiome interactions that contribute to SOC accumulation is of critical importance for bioenergy crops that are used for production of low carbon intensity biofuels, biopower and bioproducts.

Bioenergy sorghum (*Sorghum bicolor* L. Moench) is a photoperiod sensitive drought tolerant annual hybrid crop that can be used to produce biomass for forage and/or production of biofuels and bioproducts (Rooney et al., 2007; Mullet et al., 2014). Field studies and modeling showed that bioenergy sorghum harvestable biomass yields range from ~15-25 Mg/ha depending primarily on length of the growing season, abiotic stress (i.e., water supply), and soil type/profile (Byrt et al., 2011; Olson et al., 2012; Gill et al., 2014; Truong et al., 2017; Moore et al., 2021; Schetter et al., 2022). Conversion of bioenergy sorghum biomass to biofuels or biopower provides ~75-90% GHG displacement (Olson et al., 2012) at a high energy output/input (Eo/Ei) ratio (Byrt et al., 2011). A high ratio of energy produced from a bioenergy crop’s biomass, per energy of inputs, and per land area is a useful indicator of a bioenergy crop’s economic potential. Ethanol derived from bioenergy sorghum feedstocks has predicted carbon intensity (C.I.) of ~17 gCO_2_e/MJ, substantially lower than corn grain ethanol (Kent et al., 2020). Modeling indicates that production of bioenergy sorghum will have a net positive impact on soil organic carbon (SOC) (Gautam et al., 2020) consistent with long term studies showing production of bioenergy sorghum increases SOC accumulation (Shahandeh et al., 2016; Govindasamy et al., 2021). Bioenergy sorghum’s contribution to SOC can be attributed in part to long growing seasons that enable the development of large root systems comprised of up to ~175 nodal roots that reach >2 m deep in soil profiles (Lamb et al., 2022).

Sorghum produces primary roots, seminal roots, lateral roots, and three types of nodal roots (crown, brace, aerial) (Singh et al., 2010; York et al., 2022). The primary root is produced by the embryo and is the predominate conduit for water and nutrients during the juvenile stage of development. Lateral roots are generated by and emerge from root tissue (Torres-Martinez et al., 2019; Viana et al., 2022). Crown, brace, and aerial roots are nodal roots produced from stem tissue making them adventitious, however, unlike adventitious roots in tomato and other eudicots, their development is restricted to the stem-root crown and stem nodes. During early adult vegetative phase development, plants produce crown roots below ground that are closely stacked together. Brace roots formed on the next three to five above ground stem nodes penetrate the soil profile contributing to nutrient and water uptake and helping to prevent plant lodging. Nodal roots that do not reach the ground are termed aerial roots. Aerial roots, like all roots, produce root tip associated mucilage that supports a complex phyllosphere and symbiotic interactions between nitrogen-fixing microbes and roots (Werker and Kislev, 1978; Hara et al., 2019; Bennett et al., 2020).

Information about the biogenesis of bioenergy sorghum nodal root buds and the regulation of nodal root outgrowth is limited, however, extensive research on root development has been done on other plant species. These studies showed that nodal and lateral root primordia initiate from the pericycle, a thin layer of cells located between the endodermis and the phloem. A local increase of auxin primes pericycle cells for root initiation and induces expression of *WOX11*, *WOX5*, and *LBD16* leading to the formation of root primordia (Yu et al., 2017). After initiation of root primordia, proteins involved in root cell pattering (SHR-SCR) (Koizumi et al., 2012; Lucas et al., 2011; Sparks et al., 2016), maintenance of meristematic stem cells, stem cell niche/quiescence center (PLTs, LBDs) (Galinha et al., 2007) and numerous other factors help produce the root apical meristem (RAM) and associated root tissues (Torres-Martinez et al., 2019; Viana et al., 2022).

Auxin, other hormones, and signaling peptides modulate root primordia initiation, differentiation, elongation, and maturation (Casimiro et al., 2003; Overvoorde et al., 2010; Jung and McCouch, 2013; Torres-Martinez et al., 2019; Zhang et al., 2022). Once root primordia have been formed, continuous maintenance of auxin distribution is necessary to create and maintain the root quiescent center and initial cells. A signaling network comprised of mobile peptides, hormones, and protein feedback loops allow the cells in the meristem to maintain their pluripotency (Strotmann and Stahl, 2021). After the primordia have developed, they emerge from the parent tissue to become functional roots. Root emergence is stimulated by auxin and high ethylene concentrations and is repressed by ABA (Zeng et al., 2021). ABA, a key hormone involved in mediating abiotic stress responses (Danquah et al., 2014), is thought to regulate the outgrowth of nascent roots when a larger root system is needed.

Crown and brace nodal root anatomy and development have been studied extensively in maize, a C4 grass related to sorghum (Martin and Harris, 1976; Hochholdinger and Feix, 1998; Hoppe et al., 1986; Sauer et al., 2006; Taramino et al., 2007). However, information on sorghum nodal root development is limited, especially in bioenergy sorghum genotypes that grow vegetatively for most of the growing season. In a prior study, the bioenergy sorghum root system was analyzed over several seasons in the field providing a detailed description of root biomass accumulation, distribution in the soil profile, anatomy, and surface and deep lateral root gene expression (Lamb et al., 2022). In the prior study, rings of nodal root ‘buds’ were observed on the surface of the stem node pulvinus of most fully developed phytomers. Nodal root buds (NRBs) are the source of nodal roots that grow out through the epidermis later in the growing season or in response to stalk lodging. The current study characterized nodal root bud development from initiation through formation of NRBs on the surface of the stem pulvinus to better understand how bioenergy sorghum plants produce large numbers of nodal roots. This study provides a baseline of information that could be used to help optimize production of bioenergy sorghum nodal roots potentially increasing SOC and reducing the amount of nitrogen fertilizer required for crop production.

## Materials and methods

### Field plot management

The bioenergy sorghum hybrid TX08001 was planted at the Texas A&M University Farm in Burleson County, TX on 4/29/2021; plant emergence occurred 6-days after planting on 5/5/2021. Plot size was thirty-two 30 m long rows at a row spacing of 76 cm. At the time of planting, a solution of liquid ammonium polyphosphate (11-37-0), UAN 32%, and zinc sulfate was applied at a depth of 5 cm to the side of the seed for a total fertilizer yield of 45-63-0 + 5 Zn kg/ha. The soil consisted of Weswood Silt Loam (Staff, 2022). The plot was sown with enough seeds to allow thinning to 15 cm plant spacing within rows at 21 days after emergence (DAE). Seeds, before planting, were treated with Concept III^®^, a herbicide protectant, Nugro^®^ a systemic insecticide and Apron X^®^, a systemic fungicide.

### Plant tissue collection

At 120 days after emergence (DAE), five TX08001 field-grown plants were harvested for morphological analysis and RNA-seq. Leaf blades were removed for transport, and in the laboratory, leaf sheaths were dissected using a scalpel to expose the stem tissue. The relative position, number, and size of nodal root buds (NRBs) on each stem node were measured. NRB tissue samples were collected from stem nodes of phytomers 7-21 by excising a 3-4 mm deep wedge of pulvinus rind tissue containing the NRBs. Three replicate plants were sampled, and mid-internode stem rind samples from phytomers 6, 8, 9, 20, and 21, where NRBs do not form, were collected as controls. All tissue samples were flash-frozen in liquid nitrogen and stored in Whirl-Pak^®^ bags for further analysis. In addition, plants containing 7 phytomers with fully elongated internodes were harvested at 40 DAE to investigate the number of NRB rings on the nodes of plants containing less than 17 developed phytomers.

### RNA sequencing and transcriptome analysis

Tissues were ground to a fine powder with a heat sterilized mortar and pestle filled with liquid nitrogen then transferred into liquid- nitrogen chilled sterile 1.5 mL c centrifuge tubes. RNA was extracted using the Zymo RNA Mini- Prep kit. Purity and concentration of the RNA was analyzed using a Thermo ScientificTM NanoDrop One Microvolume UV-Vis Spectrophotometer before being sent for fragmentation analysis on an Agilent 5300 Fragment Analyzer using software version 3.1.0.12. RNA that passed QC was sent to the Joint Genome Institute for sequencing to a depth of 30–50 million reads. Sequenced reads were aligned to the Sorghum bicolor V3.1 genome using HISAT2 aligner (Kim et al., 2015). The transcriptome assembly and TPM normalization were conducted using StringTie version 1.3 (Pertea et al., 2015). The script prepDE.py https://github.com/gpertea/stringtie/blob/master/prepDE.py and https://ccb.jhu.edu/software/stringtie/index.shtml?t=manual was used to convert nucleotide coverage data from StringTie into read counts that were readable by differential expression statistical packages using the formula: reads_per_transcript = coverage * transcript_length/read_length. Read length was 151 bp per read. Functional annotations of the transcripts were obtained from the Sorghum bicolor V3.1 genome which is available from Phytozome 13 (McCormick et al., 2018).

### Uniform manifold approximation and projection for dimension analysis

Uniform Manifold Approximation and Projection for Dimension Reduction was used to visualize the relatedness of nodal root bud sample expression profiles (McInnes et al., 2018). Transcript counts were concatenated into gene counts and scaled across each gene. Median Absolute Deviation (MAD) scores were calculated to determine the top 1000 most variable genes which were then used for the dimension reduction analysis. This analysis was performed in *R* (4.4.0) using the library *‘*umap*’* and visualized using *‘*ggplot2*’*.

### Differential expression analysis

Differentially expressed genes (DEGs) during NRB development were identified using a two-step approach with the DESeq2 package in R, utilizing gene count data. First, NRB-specific genes, distinct from the surrounding pulvinus tissue, were identified by contrasting NRB-containing pulvinus samples from internodes 7, 8, 14, 15, 20, and 21 (combined to form group one) to mid-internode stem rind samples from internodes 6, 8, 9, 20, and 21 (combined to form group two) which lacked NRBs (control samples). Before analysis, genes were filtered based on TPM-normalized expression, retaining those with TPM > 5 in any biological replicate. Genes that were upregulated in NRBs with a fold change > 5 and an adjusted p-value < 0.05 were retained as NRB-specific genes. However, not all these genes contributed to NRB development. To identify the subset regulating NRB development, a second differential expression analysis was performed to detect DEGs between any pairwise comparison of NRB-containing stem nodes from phytomers 7-21. Genes with a fold change > |5| and an adjusted p-value < 0.05 were considered differentially expressed during NRB development. The final set of differentially expressed genes was obtained by identifying the genes differentially expressed in both datasets, resulting in 1,432 genes. This final set of genes was used for gene regulatory network (GRN) analysis.

### Gene regulatory network analysis

A multistep approach, starting from the results from the differential expression analyses, was used to calculate the GRN associated with NRB development. The input dataset into network construction was an RNA-seq, TPM normalized, dataset that spanned NRB development (which was also used above as input for one of the differential expression analyses). Using the NRB development dataset, a Weighted Gene Coexpression Network Analysis (WGCNA) was performed using the WGCNA package in R (Langfelder and Horvath, 2008). The dataset was largely unfiltered except for applying an expression threshold TPM > 5. To ensure that the network exhibited scale-free topology, the scale-free topology model fit was calculated, and the soft thresholding power that corresponded to a R^2^ closest to 0.9 was selected. A Pearson’s correlation adjacency matrix was calculated and adjusted using the soft threshold power. This adjacency matrix was used as the input to calculate the Topological Overlap Matrix to further refine the network structure. This resulting network was filtered using the common set of genes from the two differential expression analyses (1432 genes) and the mutual rank of each edge (the geometric mean of the ranking of each gene in the weight rank of the other gene of the pair) was calculated. The weight is a measure of the similarity between two genes of an edge and the mutual rank is a measure to the importance of that weight when considering the weights of all other edges that consist of one of the two genes being considered. Lasty, the network was pruned to include only edges that consisted of at least one transcription factor, or possibly two (when both genes are transcription factors), that were predicted to be capable of binding to a conserved regulatory element (CRE) in the promoter of the other gene of the edge. To facilitate TF-promoter binding prediction, the putative promoter sequences of all genes in the sorghum genome were subjected to DNA pattern analysis using the position weight matrices available in Plant Promoter Analysis Navigator–PLANTPAN3.0; (http://plantpan.itps.ncku.edu.tw/index.html) (Chow et al., 2019) to match known CREs with sequences in sorghum promoters. The input promoter sequences subjected to annotation spanned 1 kb upstream of the transcription start site. Transcription start site locations were obtained from the Morokoshi Sorghum Transcriptome Database (Makita et al., 2015). The final, unthresholded GRN can be queried using the network file provided in the supplemental materials. This final network consisted of 1431 genes because one gene did not have any edges fitting the criteria described above that would enable its retention in the network. For creation of the tables that show the first neighbor activators of, or genes activated by, the transcription factors of the network, the network was further thresholded by selecting all genes that had a weight > 0.1 and a mutual rank > 0.98.

### Gene ontology

The differential expression results were then examined for clustering to identify cohorts with distinct expression patterns. The cohorts of NRB DEGs were identified using the “wss” (within sum of square) method from the ‘*factoextra*’ package in R. The analysis indicated there were 4 major clusters of expression in the NRB DEG development dataset. K-means clustering was performed using the ‘*cluster*’ package to identify the genes within each cohort.

Genes in these NRB DEG cohorts were then sorted into functional categories using MapMan (i.e., cell wall modification, cell cycle/division, hormone metabolism/signaling, metabolism, transcription, transport, signaling) (Schwacke et al., 2019). In addition, the GO analysis program DAVID (Huang et al., 2009; Sherman et al., 2022) was used to identify ‘enriched’ functions/pathways in the four NRB development expression cohorts. DAVID uses an EASE score, a modified Fisher Exact p-value, to measure gene enrichment in annotation terms. The EASE score is a more conservative value, and terms with an EASE p-value < 0.05 were considered enriched and terms with a p-value < 0.06 and >2-fold enrichment were retained in the analysis.

### Microscopy

Vertical stem sections though NRBs were collected from stem nodes of phytomers at different stages of development for brightfield analysis and processed as previously described (Lamb et al., 2022). Samples used for analysis of general anatomy were transferred from ethanol into isopropanol in a five-step series with a 2 h incubation at each step, and then into acetone in a similar five-step series. Samples were then transferred into Spurr epoxy without an accelerant mixed 1:3 parts by weight with acetone and then allowed to incubate at room temperature for 12 h on a benchtop roller. After incubation, the samples were transferred into Spurr epoxy without accelerant mixed 1:2 parts by weight with acetone and allowed to incubate at room temperature for 12 h on a benchtop roller. After treatment, samples were transferred into Spurr epoxy without accelerant mixed 2:1 part by weight with acetone and allowed to incubate at room temperature for 12 h on a benchtop roller. The media was exchanged for pure Spurr epoxy without accelerant twice with a 6 h incubation period on a benchtop roller. Samples were then placed into Spurr epoxy with accelerant, transferred into flat bottom embedding capsules size “00” (EMS Cat# 70021 – polyethylene), and placed into an oven at 70°C for 12 h to cure the epoxy. The embedded samples were trimmed and sectioned using a Leica UC7 Ultramicrotome to a thickness of 100 nm, or gold color, using a glass knife. Sections were mounted to a chrome-alum stubbed slide and stained for 30 minutes in aqueous 0.25% (w/v) Basic Fuchsin (Millipore Sigma 857343) or FASGA as previously described (Lamb et al., 2022; Legland et al., 2017; Tolivia and Tolivia, 1987), destained in water for 5 minutes, dehydrated using a three-step series of ethanol, and placed in xylene before mounting with Permount. The mounted slides were placed in the oven at 40°C for 12 h to fully cure the Permount. Slides were visualized on a Leica DM6B light microscope, micrographs were taken with a Leica DMC 4500 5-megapixel camera, and the micrographs were processed using LAS X software from Leica. All micrographs were processed using LAS X software from Leica and GIMP 2.10.30 (GIMP team, 2019).

## Results

### Bioenergy sorghum nodal root numbers and distribution on stem nodes

**Figure 1:**
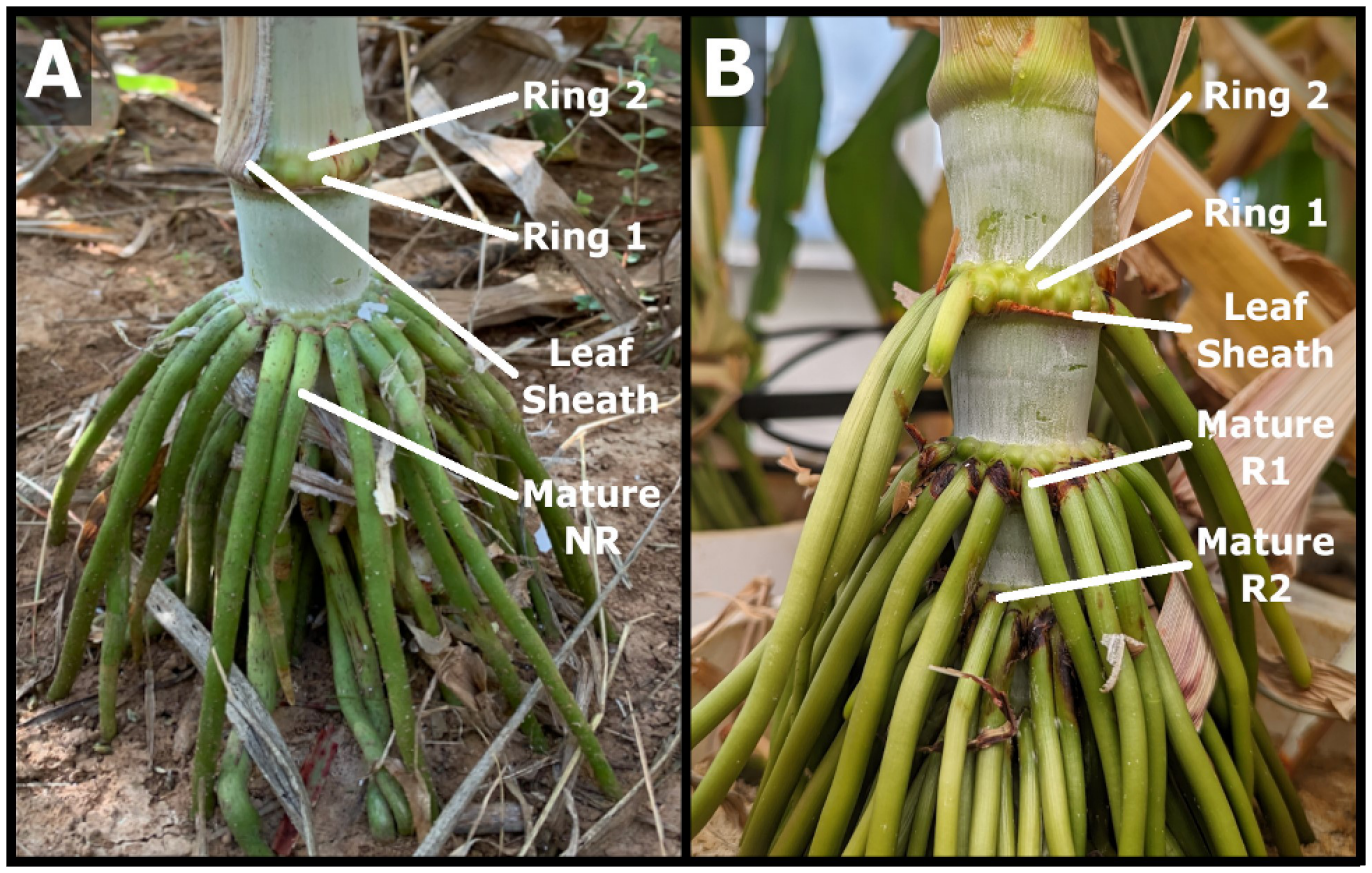
Sorghum nodal root buds and nodal root outgrowth from lower stem nodes. **(A)** Photograph of lower stem nodes (phytomers 24-27) of field grown 120 DAE TX08001each containing two rings of nodal root buds (Ring 1, Ring 2) and/or two rows of nodal roots that have grown out from the stem (NR). **(B)** Greenhouse grown TX08001 with two rings of nodal root buds (Ring 1, Ring 2) on lower stem nodes showing nodal roots emerge in a processive pattern from lowest to higher nodes. On a given node, the first ring of NRBs completely emerges before the second ring of NRBs emerges. Fully developed mature nodal roots (NR) penetrate the soil profile and proliferate further through production of lateral roots.

The number and distribution of nodal roots (NR) on vegetative field grown bioenergy sorghum hybrid TX08001 plants was examined 120 days after plant emergence. Nodal roots growing out from each stem node were tightly packed in two adjacent rows or rings that encircle the stem node pulvinus, a tissue located just above the stem nodal plexus and below the stem internode (**Figure 1A**). The first three above ground stem nodes associated with phytomers 25-27 contained an average of 20-23 nodal brace roots (range 16-29 NRs/stem node) whereas stem nodes associated with phytomers 22-24 further from the ground bore 0-10 aerial roots (**Supplementary Figure 1**). While nodes further from the ground had fewer nodal roots growing out from them, these nodes also contained numerous nodal root buds (NRBs) that were organized in two adjacent rings (**Figure 1B**). The lower ring of NRBs on a given node were larger in diameter than the upper ring of NRBs (**Figure 1B**, Ring 1 *vs*. Ring 2). Leaf sheaths on the lower nodes were brown, indicating that leaf sheath senescence occurred prior to nodal root outgrowth. The aerial portion of nodal brace roots was green and lacked lateral roots.

### Time course of NRB development

The biogenesis of the two rows or rings of NRBs on bioenergy sorghum stem nodes was investigated by taking advantage of the developmental biology of vegetative phase grass stems where the youngest nascent stem phytomers are located immediately below the stem apex and the oldest fully developed stem internode-node segments are located at the base of the stem. This developmental gradient is created by the sequential initiation of a new phytomer just below the shoot apical meristem every 3-4 days during vegetative growth. Once initiated, phytomers sequentially produce a leaf blade, leaf sheath, and fully elongated internode over the next ~18-24 days of development. The 7th phytomer below the stem apex has an internode that is nearly fully elongated (Yu et al., 2022). Bioenergy sorghum plants that are ~120 days old are composed of ~28 phytomers, where the most recently formed phytomer (P1) is located near the stem apex and P28 is located just above the crown roots.

**Figure 2:**
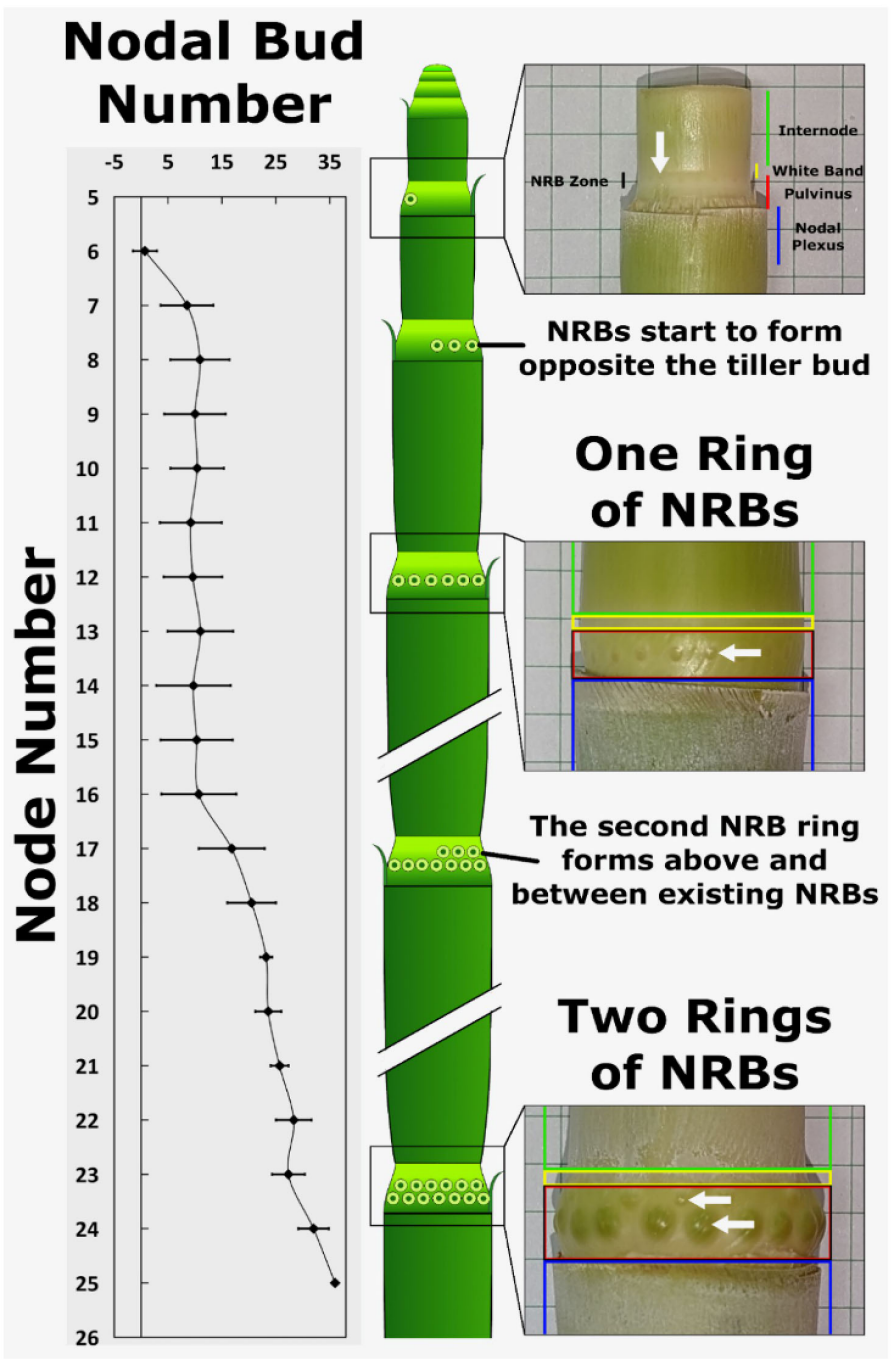
Nodal root bud proliferation during stem development. Nodal root buds (NRBs) start to accumulate in the pulvinus of the seventh node below the apical dome, initially forming on the side of the stem opposite the tiller bud. The first ring of 10-15 nodal root buds is completed on the 9^th^ node. A second ring of nodal root buds starts forming on the 17^th^ node opposite the tiller bud and above the first ring of nodal root buds. The number of buds in the second ring increases to 25-30 by node 21-24. A diagram showing the time course of NRB accumulation on the stem node pulvinus is shown (center).

NRB formation occurs approximately ~4 mm below the upper end of the stem pulvinus, a specialized stem tissue located above the nodal plexus and below the intercalary meristem/internode tissue (Yu et al., 2022). To determine when NRBs form during phytomer development, the number and distribution of NRBs on stem nodes of each phytomer of 120-day-old TX08001 plants was quantified (**Figure 2**). NRBs were not observed on stem nodes of the first six phytomers located immediately below the shoot apex. Nascent NRBs were first detected as small white dots in the rind of the pulvinus of the 7th node below the shoot apex (**Figure 2**). Nascent NRBs initially form on the side of the stem furthest from the tiller bud, which is located at the base of the pulvinus in the leaf axil, just above where the leaf sheath tissue joins the stem (Yu et al., 2022). The appearance of nascent NRBs on the pulvinus of the 7th node occurs when the growth of stem internode tissue located above the pulvinus is nearly complete and the activity of the intercalary meristem is decreasing (Yu et al., 2022).

Additional NRBs form on the same node during the next 6 to 8 days eventually forming a complete ring of ~12 nascent root buds around the pulvinus. At this point in development, phytomer 7, where the onset of the first ring of NRBs was detected, is now the 9th phytomer below the apex. No further increases in NRB number were observed from node 9 to 16, a period of development corresponding to ~24-32 days. NRB diameters increase to a small extent during this phase of development, although precise measurement of the increase in diameter was difficult to assess due to the diffuse nature of the bud-stem tissue boundary. Starting with the 17th node, a second ring of NRBs begins to accumulate on the side of the pulvinus opposite the tiller bud and above the first ring of NRBs, and by the 21st node, the total number of NRBs per node reached 25-30 (**Figure 2**). At this stage of development, both rings of NRBs were visible, however, NRBs in the basal ring that formed first were larger in diameter and darker in color. The diameter of the first ring of NRBs increased steadily over the next 15-20 days of development prior to the onset of nodal root outgrowth (**Figure 2**). Nodal root outgrowth occurred from the lower ring of NRBs after both rings of NRBs were visible. Outgrowth of the upper ring of NRBs was delayed relative to the outgrowth from the first ring of NRBs.

Examination of 40-day old TX08001 plants at the 14-leaf stage showed that stem nodes of phytomers 1-6 lacked NRBs, and stem nodes of phytomers 7 and 8 contained only one ring of NRBs. This is consistent with the finding that the second ring of NRBs forms later in plant development when stems are comprised of at least ~17 phytomers.

### NRB development morphology analysis

**Figure 3:**
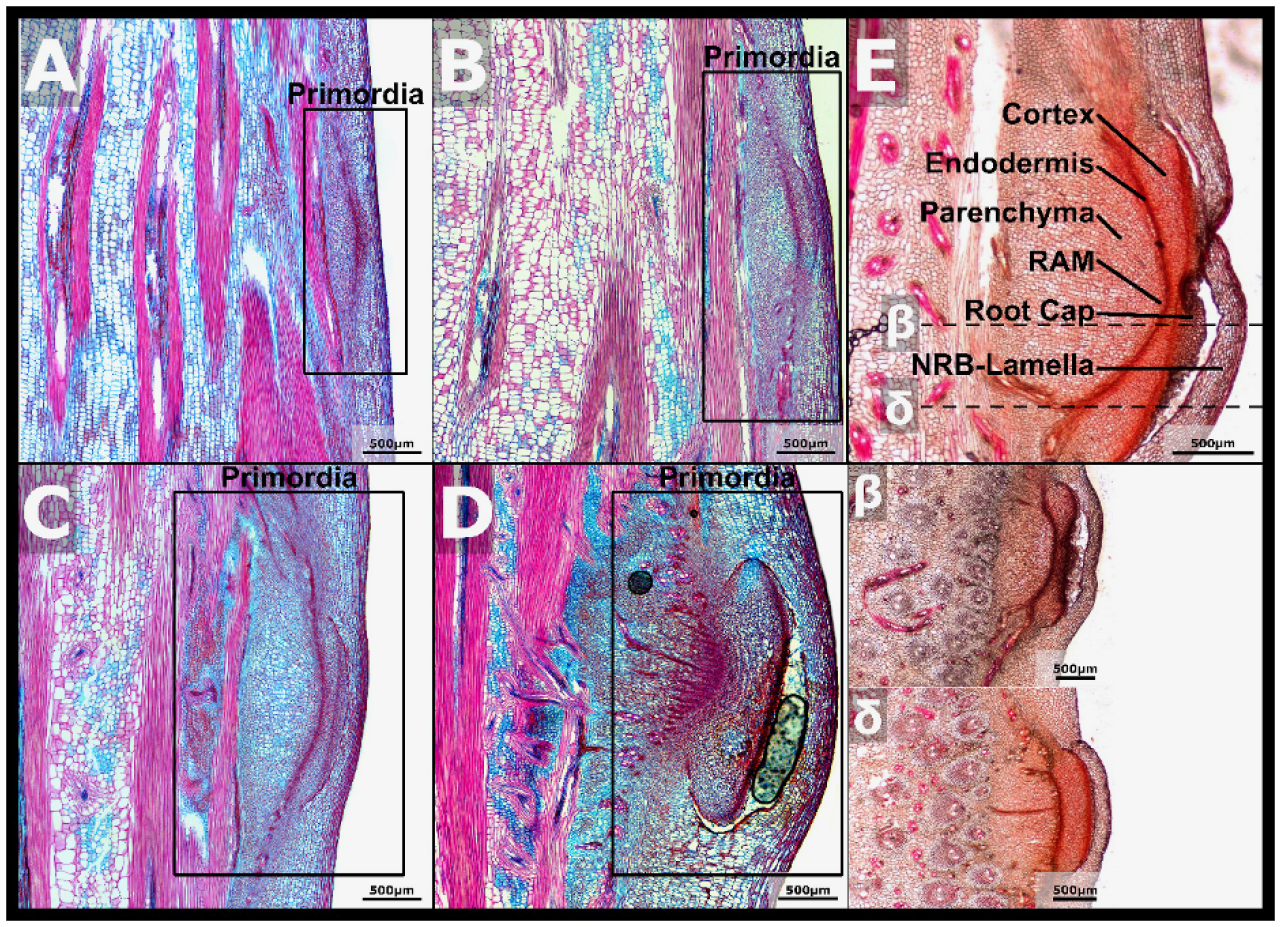
Anatomy of nodal root buds during development. **(A)** A FASGA stained section of node seven with the primordia highlighted. **(B)** A FASGA stained vertical section of node eight with the NRB primordia highlighted. **(C)** A FASGA stained section of node 16 with the NRB primordia highlighted. **(D)** A FASGA stained section of a more developed NRB. **(E)** Basic Fuchsin stained sections of fully developed NRBs with the cortex, endodermis, parenchyma, RAM, and root cap highlighted. β is a section just below center of the nodal bud, and δ is a section near the base of the bud.

Micrographs of NRB development taken from stem nodes of increasing age showed that the size and morphological complexity of NRBs increased during development (**Figure 3**). Early stage nodal root primordia were comprised of clusters of small blue and purple staining cells adjacent to vascular bundles (**Figure 3A**, boxed region, nascent NRB primordia). Late in NRB development, just prior to NR emergence, the NRB endodermis, cortex, nascent root cap, RAM and lamellae could be identified in micrographs (**Figure 3E**). The cellular structures observed in fully developed NRBs were traced back to earlier stages of development to better understand the timing of their biogenesis. For example, the NRB endodermis, which plays a role in the formation of the epidermis and root cap of maize lateral roots (Torres-Martinez et al., 2019), was observed in micrographs of nascent NRBs forming on the 7/8th, 16th, and older nodes (**Figures 3A-E**). The cortex, a tissue that develops between the endodermis and stem epidermis, was tentatively identified in micrographs of NRBs on the 16th node (**Figure 3E**). The overlaying stem epidermis and the NRB lamella protect the nascent NRBs from biotic and abiotic stresses such as desiccation and herbivory. A space was observed between the NRB lamellae and the bud cortex later in NRB development (**Figures 3D, E**). This space could be the result of hydrolysis of cells or cell walls located between the cortex and LRB lamellae prior to nodal root outgrowth.

### NRB development transcriptome analysis

Genes involved in NRB biogenesis were identified by collecting transcriptome profiles of NRBs at different stages of development from phytomers 7-21. UMAP analysis of NRB transcriptome data showed that NRB biological replicates derived from each stem node clustered together and that NRB transcriptome profiles collected from phytomers of increasing age changed in a continuous fashion as a function of NRB development (**Supplementary Figure 2**). PCA analysis of NRB development transcriptome profiles and profiles from stem internode tissue lacking NRBs generated separate clusters as expected since the NRB development program is a specialized transcriptome program that differs from the stem internode development program (**Supplementary Figure 3**).

Genes differentially expressed in developing NRBs were identified by comparing transcriptome profiles from NRB tissue excised from the rind of the stem pulvinus of nodes 7, 8, 14, 15, 20 and 21 to profiles of rind tissue from stem internodes, a tissue that does not form NRBs, from phytomers 6, 8, 9, 19, 20. This comparison identified 2,033 genes that are expressed at higher levels in NRB rind tissue compared to internode rind tissue (>5 TPM, >5-fold DE, adjusted p-value <0.05) (**Supplementary Table 1**). Approximately 4,976 genes were differentially expressed in NRB tissue collected from phytomers 7-21 (>5 TPM, >5-fold DE, adjusted p-value <0.05) (**Supplementary Table 2**). The intersection of these two sets of NRB DEGs identified 1,432 genes that were both differentially expressed in NRB tissue (*vs*. internode rind tissue) and differentially expressed during NRB development (**Supplementary Table 3**).

Visual inspection of NRB DEG expression during NRB development revealed several patterns (high to low, low to high, etc.). Clustering analysis indicated 4 major clusters in the NRB DEG development dataset. The four expression patterns were visualized by plotting the median expression values at each time point in development for each of the four expression cohorts (**Supplementary Figure 4**). The four DEG expression cohorts had the following patterns of expression; (i) elevated expression early in NRB development (P7-9) followed by lower expression later in NRB development (**Supplementary Table 4**, Early NRB DEGs), (ii) high expression early in NRB development (P7-9), then lower expression (P10-P16), followed by increased expression in NRBs from older nodes (P20-21) (**Supplementary Table 5**, Early & Late NRB DEGs), (iii) elevated expression during mid-NRB development (P10-16) (**Supplementary Table 6**, Mid-NRB DEGs), and (iv) low expression early in NRB development followed by increased expression during NRB development to a maximum late in NRB development (**Supplementary Table 7**, Late NRB DEGs). Genes in each of these NRB DEG cohorts were sorted into functional categories using MapMan (i.e., cell wall modification, cell cycle/division, hormone metabolism/signaling, metabolism, transcription, transport, signaling) (Schwacke et al., 2019) (**Supplementary Tables 4**-**7**). In addition, the GO analysis program DAVID (Huang et al., 2009; Sherman et al., 2022) was used to identify ‘enriched’ functions/pathways in the four NRB development expression cohorts (**Supplementary Table 8**). GO analysis showed that the NRB Early-DEGs were enriched in genes involved in cell growth, morphogenesis, cell wall biogenesis, fatty acid biosynthesis and responses to oxidative stress (**Supplementary Table 8**). MapMan analysis of this same group of genes identified numerous sorghum homologs of xyloglucan endotransglucosylase/hydrolases (XTHs), expansins (EXPs) and pectin-lyases but no genes involved in secondary cell wall biosynthesis (**Supplementary Table 4**). NRB DEGs with high expression early and late in NRB development were highly enriched in genes involved in cell proliferation, cell cycle, cell division, and DNA replication (**Supplementary Tables 5**, **8**). The Early- and Mid-NRB DEGs (**Supplementary Tables 4**, **6**, **8**) were enriched in genes involved in lipid and wax biosynthesis, genes encoding GDSL proteins, and genes that contribute to stress tolerance. The Late-NRB DEGs were enriched in genes involved in cellular morphogenesis, transmembrane transport, lipid oxidation/oxylipin biosynthesis and defense responses (**Supplementary Tables 7**, **8**).

### Hormone regulation of NRB development

Hormones play an important role in root initiation and development (Yamoune et al., 2021; Bassuner et al., 2007). Numerous genes involved in auxin (IAA), cytokinin (CK), gibberellin (GA), brassinosteroid (BR), strigolactone (SL), ethylene, and ABA metabolism were expressed during NRB development as well as genes involved in IAA-signaling (*ARF3, ARF5, ARF10*), CK-signaling (*MYB3R-1, MYB3R-4, TCX2, WEE1*) and GA-signaling (*AN3, GRFs, LUG, ORE15, bHLH093, SPL9)* (**Supplementary Table 9**). Expression of many of the hormone pathway genes was high early in NRB development, then decreased during mid-NRB development before increasing again later in NRB development, an expression pattern also observed for genes involved in cell proliferation (**Supplementary Table 5**).

IAA transport plays a key role in root initiation and other steps in root development (Steffens and Rasmussen, 2016; Torres-Martinez et al., 2019; da Costa et al., 2020). Three types of sorghum IAA transporters were differentially expressed during sorghum NRB development including two sorghum homologs of *AtLAX2* that encode auxin importers (**Supplementary Table 9**) (Péret et al., 2012). A gene encoding SbPIN4, an auxin exporter involved in polar auxin transport, also showed relatively high expression early in NRB development and a second peak of expression late in NRB development (**Supplementary Table 9**). Expression of a gene encoding the auxin exporter SbABCB19 was elevated throughout NRB development (**Supplementary Table 9**). A second gene encoding SbABCB19 and SbPIN2 showed increasing expression late in NRB development.

### NRB development

Information about the potential role of NRB DEGs was obtained by identifying sorghum homologs of genes previously shown to play a role in root development in other plant species (i.e., TAIR database; (Torres-Martinez et al., 2019; Kortz et al., 2019; Meng et al., 2019). The expression patterns and predicted roles of this group of sorghum homologs in NRB development are described below.

### NRB initiation (PFA5, WOX4, NGAL2, ANT, FAS2)

**Figure 4:**
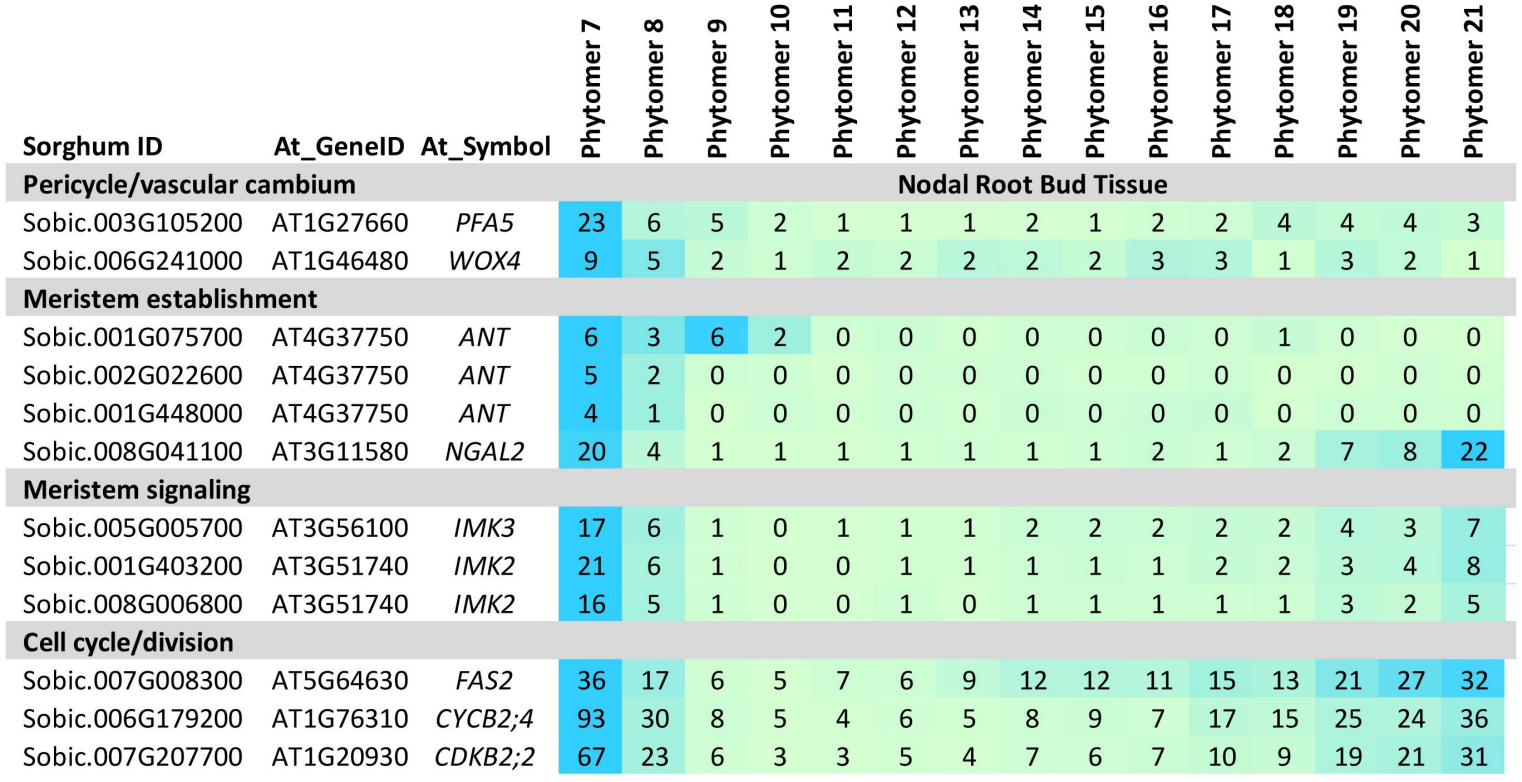
Expression of genes involved in nodal root bud initiation. Genes involved in nodal root bud initiation (*PFA5, WOX4, ANT*) meristem activity and signaling (*NGAL2, IMK*) and cell proliferation (i.e., cyclins) are expressed at high levels early in nodal root bud development. Numeric data represents TPM normalized expression. Each data point is the mean of three biological replicates. In the heat map, blue represents higher expression, green represents lower expression. Phytomer/stem node 7-21 indicates the stem node from which NRBs were collected for transcriptome analysis.

*WOX4*, a gene involved in meristem maintenance in the vascular cambium (Zhang et al., 2019; Kucukoglu et al., 2017), primary roots (Chen et al., 2020), and adventitious roots (Wang et al., 2020), was expressed early in NRB development (**Figure 4**). A sorghum homolog of *PFA5*, a gene expressed in Arabidopsis lateral root apical meristem xylem precursor cells (Zhang et al., 2021), was also differentially expressed early in NRB development (**Figure 4**). Other genes expressed early in NRB development included three sorghum homologs of *ANTEGUMENTA* (*ANT*), key regulators of organ initiation, meristem identity and development (Krizek et al., 2021), *NGAL2*, a regulator of meristem activity (Nicolas et al., 2022), and genes encoding IMK meristem signaling kinases and meristem REM TFs (Mantegazza et al., 2014). In addition, *FAS2*, a gene involved in RAM cell cycle regulation (Abe et al., 2008; Viana et al., 2022) and numerous genes involved in the cell cycle, cell division, DNA synthesis, histone synthesis, and cytokinesis were expressed at high levels early in NRB development with a second peak of expression later in development (**Figure 4**, **Supplementary Table 4**).

### NRB development (WOX11/5, PLETHORAs, LBDs, LRP1, SMB, RGFs)

**Figure 5:**
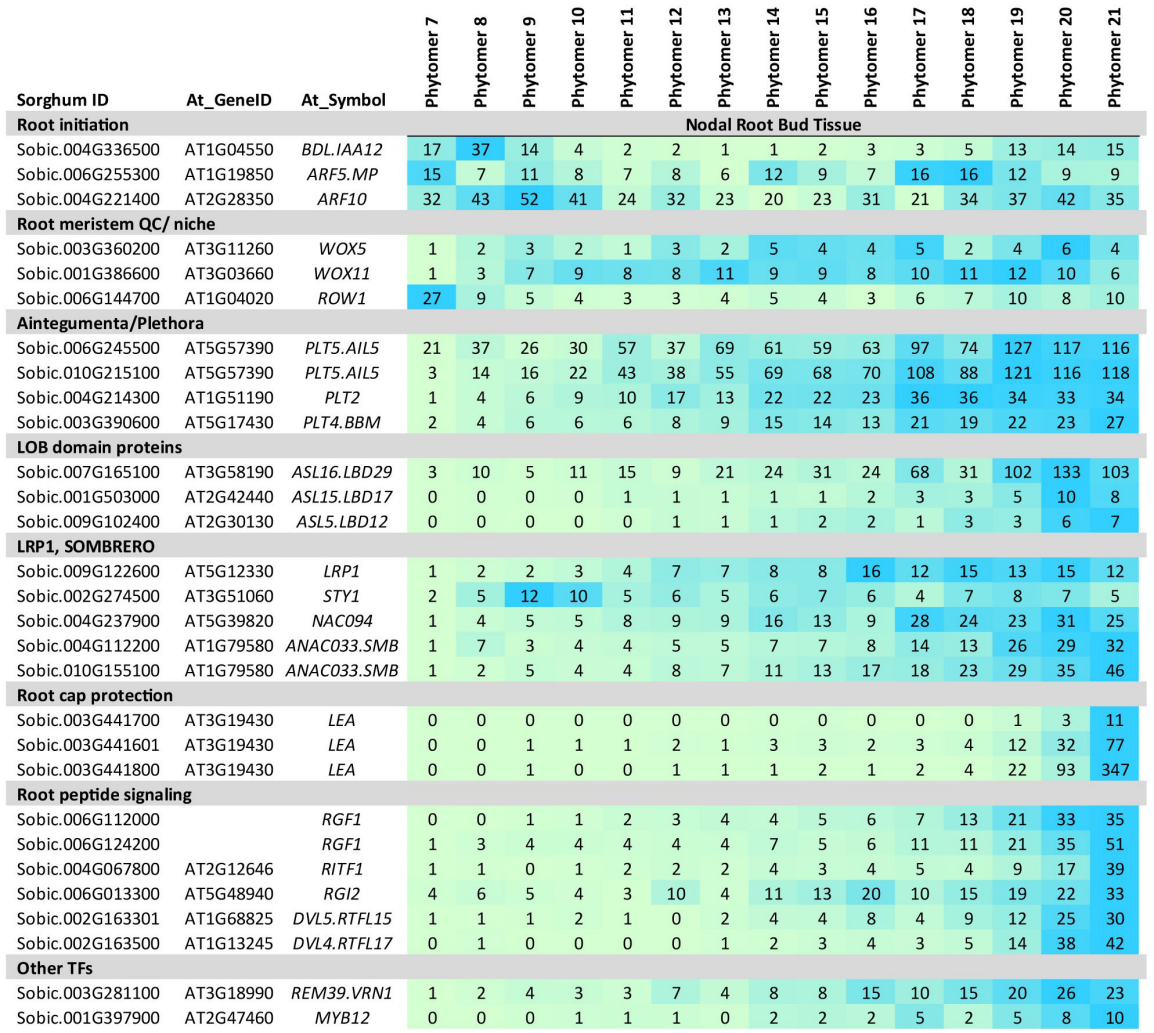
Expression of genes involved in nodal root bud development. Sorghum homologs of genes involved in auxin signaling (*ARF, IAA, LBDs, LRP1*), root development (*WOX11, WOX5, PLTs, LBDs*) and root cap formation (*SMB, RITF1*) are differentially expressed during NRB development. Numeric data represents TPM normalized expression. Each data point is the mean of three biological replicates. In the heat map, blue represents higher expression, green represents lower expression. NRB development spans phytomer/stem node 7-21.

Formation of lateral or adventitious root primordia is a multistep process involving establishment of root cell identity, formation of the stem cell niche (SCN)/quiescence center (QC) and root cap tissue prior to root outgrowth (Torres-Martinez et al., 2019). A sorghum homolog of *BDL.IAA12*, a regulator of primary root initiation (Herud et al., 2016), was expressed early in NRB development (**Figure 5**). BDL.IAA12 binds to and represses ARF5.MP, a transcription factor involved in primary root initiation (Herud et al., 2016). A sorghum homolog of *ARF5.MP* was expressed throughout sorghum NRB development. *WUSCHEL RELATED HOMEOBOX* (*WOX*) genes play key roles in the establishment and maintenance of the quiescent center (QC). The expression of sorghum homologs of *WOX11* and *WOX5* increased by P9 and was maintained thereafter, consistent with their role in root meristem QC establishment and maintenance (**Figure 5**). In a previous study, *WOX11/12* was found to activate *WOX5* to promote root initiation in Arabidopsis (Hu and Xu, 2016). *ROW1*, a gene that helps confine *WOX5* expression to the QC (Zhang et al., 2015), was expressed at high levels in P7 followed by decreased expression until late in NRB development (**Figure 5**).

Sorghum homologs of *PLT2*, *PLT4*/*BBM* and *PLT5* showed increasing expression during NRB development (**Figure 5**). These AIL transcription factors play various roles in root development and are regulated by IAA/auxin signaling in a complex way (Horstman et al., 2014). PLT2 and BBM (PLT4) are key regulators of root identity and root meristem niche formation and maintenance (Horstman et al., 2014).

Genes encoding LOB-domain (LBD) proteins play key roles in lateral root formation in Arabidopsis and rice (Cho et al., 2019; Lee et al., 2009). In rice (Inukai et al., 2005) and maize (Taramino et al., 2007), mutation of *LBD29.CRL1* results in loss of crown roots. In the current study, expression of sorghum homologs of *LBD12*, *LBD17* and *LBD29* increased to a maximum late in NRB development (**Figure 5**). A previous study showed that auxin signaling that involves LBD29 represses NAC regulators that induce secondary cell wall synthesis on vascular bundle fiber cells (Lee et al., 2019). Genes involved in secondary cell wall formation (Hennet et al., 2020) were not differentially expressed during NRB development.

*LATERAL ROOT PRIMORDIUM1* (*LRP1*) is expressed in the root meristems of lateral and adventitious roots (Smith and Fedoroff, 1995; Singh et al., 2020). LRP1 can interact with other members of the SHI/STY protein family to modulate/inhibit root lateral root development (Singh et al., 2020). Sorghum homologs of *LRP1* and *SHI/STY1* were both expressed during NRB development (**Figure 5**).

The expression of two sorghum homologs of *SOMBRERO* increased during NRB development (**Figure 5**). SOMBRERO regulates stem cell activity and determines root cap organ size in conjunction with FEZ by modulating programmed cell death (Fendrych et al., 2014; Willemsen et al., 2008). Expression of the *SbSMB1* genes later in NRB development but prior to root outgrowth is consistent with morphological analysis showing the appearance of the root cap late in NRB development. In contrast, FEZ expression was below the TPM threshold used in this study. Increased expression of root cap LEAs that protect cells from dehydration damage was also observed late in NRB development (Candat et al., 2014) (**Figure 5**).

The ROOT MERISTEM GROWTH FACTORs (RGFs) are peptides expressed in root growing zones that interact with receptors such as RIF1 and RGI and regulate *PLT* expression (Matsuzaki et al., 2010). Sorghum homologs of genes encoding RGF peptides and RIF1/RGI receptors were differentially expressed during sorghum NRB development (**Figure 5**) indicating these regulators of root meristem activity by modulate NRB development. Several homologs of genes encoding DVL peptides showed a similar pattern of expression during NRB development.

### NRB gene regulatory network analysis

**Figure 6:**
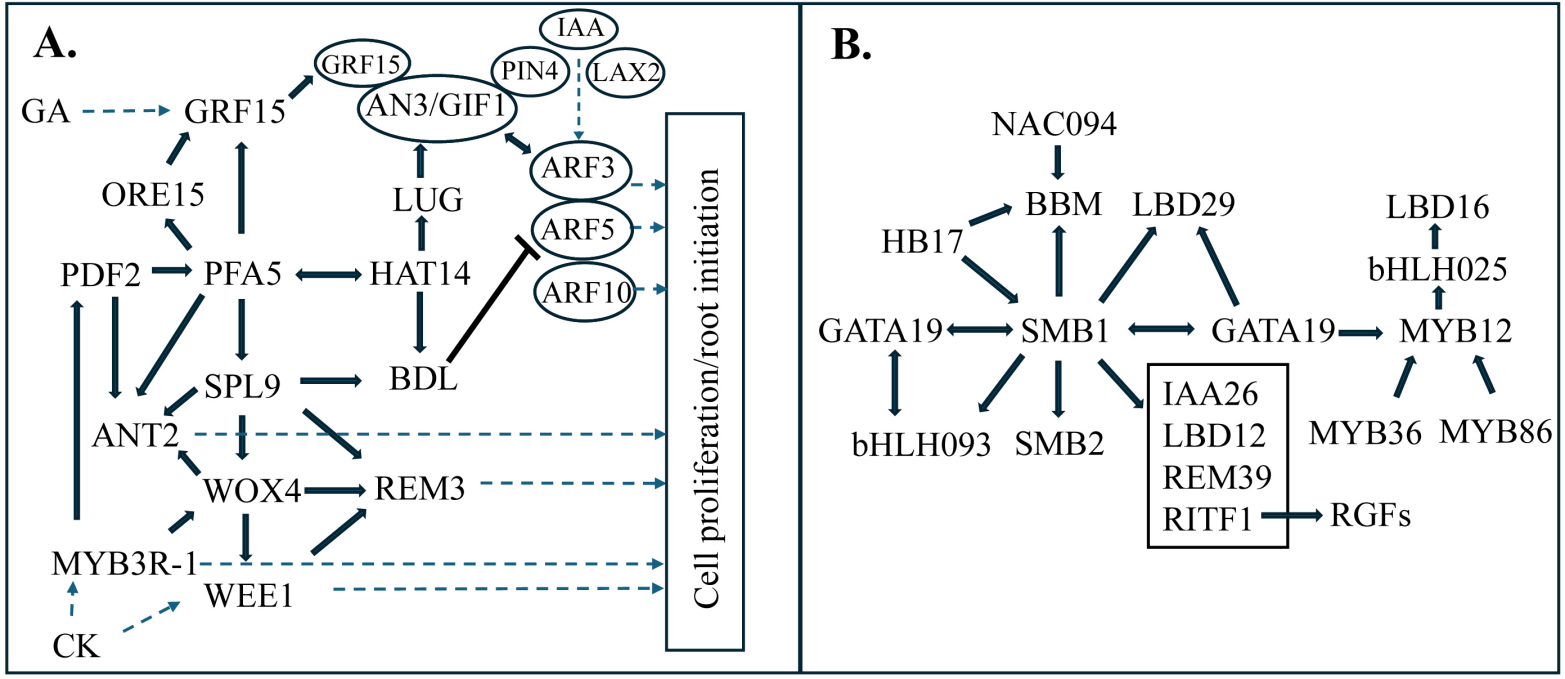
Nodal root bud gene regulatory network transcription factor modules. Transcription factor (TF-TF) interactions predicted by gene regulatory network analysis revealed modules expressed early **(A)** and Late **(B)** in NRB development. **(A)** GRN sub-module centered on PFA5, a transcription factor expressed in the pericycle, that is connected to genes involved in hormone signaling (GA, CK, IAA) and cell proliferation/root initiation. **(B)** GRN sub-module centered on SMB1 (SOMBRERO) a transcription factor involved in formation of root columella/cap cells.

Variation in gene expression during organ, tissue, or cell development provides information useful for co-expression and gene regulatory network (GRN) analysis (Yu et al., 2022; Chemelewski et al., 2023). In other plant systems, GRNs were found to integrate environmental sensing and the growth of the root cambium (Hoang et al., 2020). In the current study, to better understand how NRB DEGs are regulated during development, GRN analysis was carried out using methods that combine co-expression analysis (WCGNA) and information that connects genes encoding TFs (hubs) to TF binding sites in promoters of target genes (edges) (**Supplementary Figure 5**). Each TF in the network is potentially connected to and could regulate the expression of other TFs (**Supplementary Data Sheet 1**). Most of the edges or connections in the GRN were based on positively correlated co-expression consistent with TF activation of target genes. The NRB GRN contained a group of TFs that are expressed at high levels early in NRB development, followed by lower expression and a second increase in expression in NRBs from older stem nodes (**Figure 6A**; **Supplementary Data Sheet 3**). TFs in this module are connected to genes involved in cell proliferation (cell cycle, cell division, DNA synthesis, etc.) (**Figure 6A**, right). This early development NRB GRN module included *PFA5* and *WOX4*, TFs expressed in the vascular bundle pericycle and cambium, respectively. The ‘PFA5’ NRB GRN module also contained genes encoding MYB3R-1, SbGRF15 and BDL.IAA12 potential enabling GA, CK and IAA signaling and regulation of cell proliferation through modulation of *GRF15* expression and AN3 activity and *ANT*, *BBM*, and *REM3* expression (Yu et al., 2022; Wang et al., 2018).

Another group of TFs that increased in expression later in NRB development formed a separate GRN module that included *SOMBRERO* (*SMB*) (**Figure 6B**; **Supplementary Data Sheet 4**). Sorghum homologs of *SMB* (*SbSMB1, SbSMB2*), *GATA19*, *BBM*, and *LBD29* were included in this NRB GRN module as well as *bHLH093*, a gene involved in meristem function (Poirier et al., 2018). A gene encoding RITF1 which has an important role in RGF1 signaling (Yamada et al., 2020), was regulated by TFs in the ‘SMB’ GRN module.

### Putative regulators of NRB growth/outgrowth

NRB outgrowth is inhibited during the long duration of NRB development on stem nodes. Variation in hormone levels or signaling could regulate NRB outgrowth. Genes involved in GA-stimulated cell proliferation such as *SbGRF15* and *SbAN3* are expressed early in NRB development, however, four genes encoding GA2 oxidases that are involved in GA metabolism are also expressed in NRBs (**Supplementary Table 8**). Elevated levels of ABA or ABA-signaling are often associated with growth inhibition and acquisition of abiotic stress tolerance. Expression of *TINY*, an ABA regulated AP2-domain TF that represses growth (Wei et al., 2005) was elevated soon after NRB initiation through late-NRB development (**Supplementary Table 10**). *ATH13* genes that contribute to abiotic stress tolerance were also expressed at relatively high levels through mid-NRB development (**Supplementary Table 10**). Furthermore, several genes that are involved in ABA-signaling (*EEL, ATHB17, ATHB21, ATHB40*) were expressed during NRB development (**Supplementary Table 10**). ATHB21, ATHB40, and ATHB53 were previously associated with repression of axillary bud outgrowth (Gonzalez-Grandio et al., 2017). A gene encoding HK5, a regulator of CK levels, was induced later in NRB development (**Supplementary Table 10**). Expression of *AP2* and *FUL* in NRB tissue could indicate these TFs regulate meristem activity based on their proposed role in mediating meristem arrest (Martinez-Fernandez et al., 2020; Wang et al., 2023; Merelo et al., 2022).

## Discussion

The long duration of bioenergy sorghum’s adult vegetative phase typically results in production of 4-5 m long stems comprised of >40 phytomers and >40 stem node-internode segments of varying age (Olson et al., 2012; 2013). Approximately ~175 nodal (crown, brace, aerial) roots emerge either below ground from the stem-root crown or from above ground stem nodes during a ~150-200 day growing season (Lamb et al., 2022). Above ground stem nodes that are close to the soil produce 20-30 large diameter nodal roots that enter the soil profile helping to prevent shoot lodging and increasing the root systems’ capacity for water and nutrient uptake. The aerial portion of ground penetrating nodal roots and aerial roots that never reach the ground associate with nitrogen fixing diazotropic bacteria that contribute to plant N-requirements (Mechan-Llontop et al., 2022). These attributes of bioenergy sorghum’s root system are critically important for sustainable accumulation of biomass needed for production of low carbon intensity biofuels and bioproducts (Lamb et al., 2022). Therefore, a deeper understanding of nodal root biogenesis could contribute to further improvement in bioenergy sorghum resilience and sustainability. The current study found that sorghum NRBs are produced at two different stages of adult vegetative-phase stem growth. When adult vegetative phase plants contain 8 phytomers, an initial ring of ~10-15 NRBs begins forming on the 7^th^ phytomer below the shoot apex with complete rings formed by the 9^th^ phytomer. In older vegetative plants, a second ring of NRBs begins to form on the 17^th^ phytomer below the apex. The addition of a second ring of NRBs to older stem nodes as plants grow larger may reduce plant lodging (brace roots) and increase aerial root production.

Nodal root bud development was analyzed by collecting rind tissue containing NRBs from the stem nodes of 120-day-old vegetative phase plants. The shoots of these plants were comprised of ~25-30 phytomers of increasing age as a function of distance from the shoot apex. Microscopic analysis of NRB anatomy and transcriptome analysis was conducted from nodal root bud initiation on the 7^th^ phytomer below the shoot apex through the development of the second rind of NRBs (17^th^-21^st^ node) but prior to NR emergence. NRB development can span up to ~40 days or more before outgrowth. In contrast, the time from lateral root initiation to emergence ranges from 1.6-3.6 days (Torres-Martinez et al., 2019). The long duration of NRB development on above ground stem nodes results in the formation of large diameter (up to 5 mm) buds, aerial and ‘brace’ roots (Lamb et al., 2022). Since maize and sorghum roots do not increase significantly in diameter via secondary growth (Hochholdinger et al., 2004), the long duration of NRB development may aid production of numerous large diameter brace roots required to support 4-5 m tall plants with large canopies later in the growing season.

Nodal root emergence from a specific stem node occurred initially by outgrowth from the lower ring of NRBs and later from the younger upper ring of NRBs. The delayed timing of nodal root outgrowth from the upper ring of NRBs may enable plants to adapt to changing environments that occur following outgrowth of the first ring of NRBs. NRB’s formed later in development on stem nodes located higher on the stem often form aerial roots that do not reach the ground. Mucilage covered large diameter aerial roots harbors a complex phyllosphere (Mechan-Llontop et al., 2022) that includes N_2_-fixing bacteria (Hara et al., 2019). In addition, nodal roots will grow out from NRBs formed on many upper stem nodes if sorghum plants become lodged during the growing season.

### NRB localization, number, and developmental time course

Roots that grow out from stem nodes are unique to monocot species (Hochholdinger et al., 2004). Stem nodes are comprised of a nodal plexus, where the leaf sheath joins the stem, and a pulvinus, a short, specialized tissue located immediately above the nodal plexus and below the internode (Clore, 2013; Yu et al., 2022). NRBs form approximately 4 mm below the intercalary meristem in the rind of the pulvinus. The stem intercalary meristem, a hallmark of monocot stems, generates internode tissue between nodes (McKim, 2020). Recent analysis of the sorghum intercalary meristem revealed that this specialized region of cell proliferation is localized at the upper end of the pulvinus (Yu et al., 2022). The pulvinus is characterized by elevated expression of *SbLOB*, a gene associated with boundary zones that are sites of meristematic activity (Wang et al., 2016). The localization of the intercalary meristem at the upper end of the pulvinus, tiller buds at the lower end of the pulvinus, and NRBs within the pulvinus is consistent with the designation of the pulvinus as a boundary zone (Yu et al., 2022). The onset of NRB initiation in the rind of the pulvinus of phytomer 7 is noteworthy, since this occurs after tiller buds are formed and when the activity of the intercalary meristem at the upper end of the pulvinus is rapidly decreasing (Yu et al., 2022).

Sorghum stem nodal root buds were first detected as small white dots on the pulvinus of the 7th stem node below the shoot apex on the side of the stem opposite the tiller bud. The initial repression of NRB formation on the side of the stem closest to the tiller bud may indicate that tiller buds directly or indirectly inhibit the establishment of auxin maxima required for nodal root bud initiation. By node 9, ~6-8 days after initial visualization of the first NRBs, a ring of 10-15 white dots marking sites of NRB initiation encircles the stem. The number of NRBs remained constant from stem node 9-16 (~24 days of development). Visual inspection of node 17 revealed that a second ring of NRBs was forming immediately above the first ring of NRBs. The second rind of NRBs is initiated on the side of the stem away from the tiller bud as occurs during formation of the first ring of NRBs. Formation of the second ring increases the total number of NRBs/node to ~25 by node 21. It is not clear what triggers the formation of the second ring of NRBs although it was noted that leaves/leaf sheaths of node 17 are well below the top of the canopy and beginning to senesce (Olson et al., 2012). Shading due to the continuous production of leaves by the shoot apex causes the senescence of leaves lower in the canopy which alters the flow of sugars, auxin, and other signals from leaves to the stem. Senescence of the leaf sheath also exposes the pulvinus to more direct light and minimizes the barrier to nodal root outgrowth. Two genes involved in NPH3-light signaling increased in expression later in NRB development coincident with formation of the second ring of NRBs (**Supplementary Tables 7**, **8**).

### NRB development transcriptome dynamics

**Figure 7:**
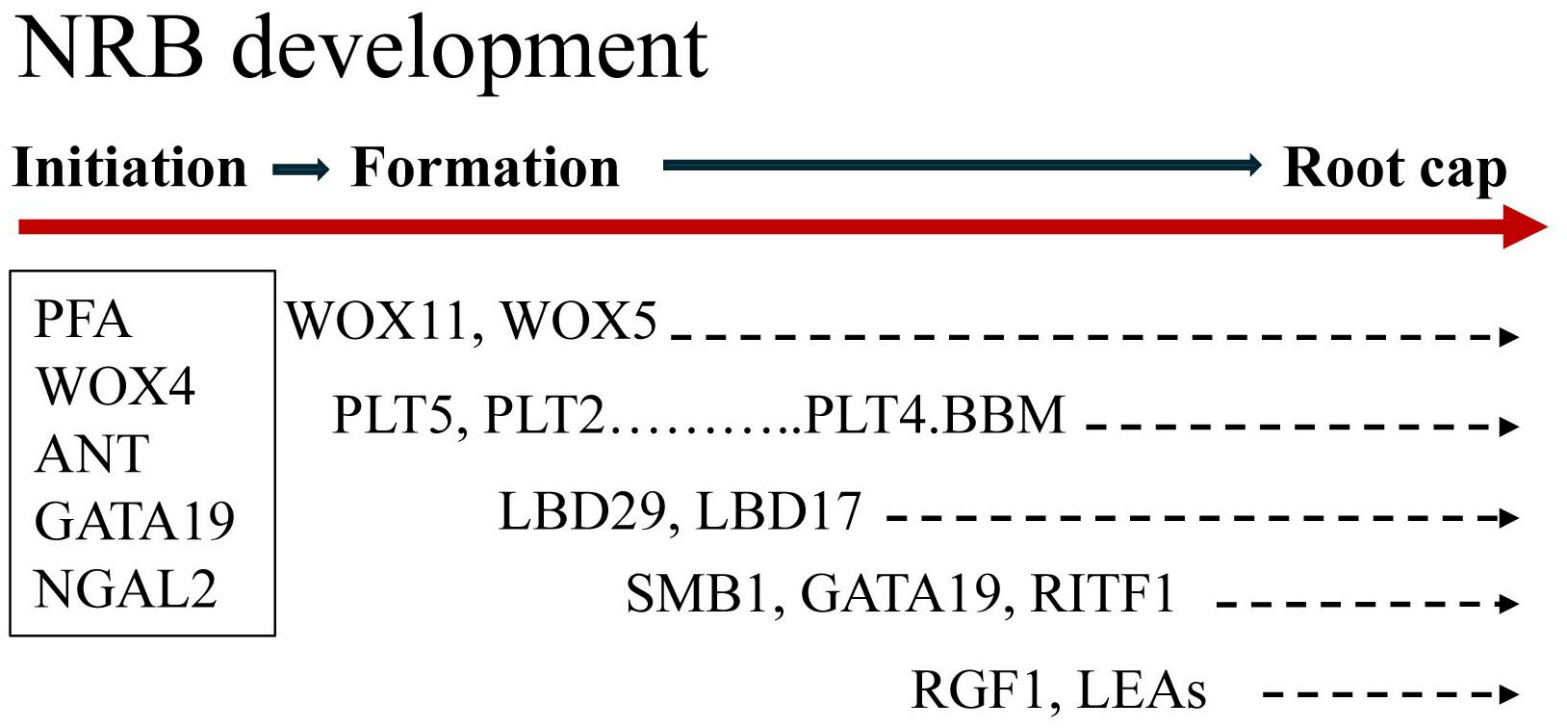
Overview of sorghum nodal root bud (NRB) development. NRB initiation in the pulvinus of phytomer 7 occurs in a sub-epidermal tissue that shows elevated expression of genes encoding the auxin transporters PIN4, ABCB1 and LAX2 and the genes *PFA5*, *WOX4, ANT, GATA19* and *NGAL2* that are predicted to modulate hormone-modulated cell proliferation. Expression of *WOX11* and *PLT5* occurred early in NRB formation followed by induction of *PLT5/2/4* and *LBD29/17* and *RGF*s. Expression of *SMB*1, *GATA19, RIFT1, RGF1* and *LEA*s occurred later in NRB development when the root cap was forming. The duration of NRB development from initiation to outgrowth can take ~40 days, however, the timing of nodal root outgrowth varies depending on plant development and environmental conditions.

Transcriptome analysis identified cohorts of genes expressed at different times during NRB development and generated information useful for gene regulatory network analysis. **Figure 7** provides an overview of sorghum NRB development showing when key genes are expressed during the developmental process. Approximately 1,500 genes were differentially expressed during NRB development. Four cohorts of genes with different patterns of expression were identified; (1) genes expressed at high levels only early in NRB development, (2) genes expressed at high levels early in NRB development followed by a decrease in expression and a subsequent increase in expression later in NRB development, (3) genes expressed highest during mid-NRB development, and (4) genes showing a steady increase in expression to a maximum later in NRB development. Some of the genes expressed at high levels only early in NRB development could be involved in differentiation of the epidermis and rind since the wedges of tissue that contain NRBs used for transcriptome analysis included epidermal and sub-epidermal cell layers. Genes with high expression early in NRB development followed by lower expression and a second rise in expression starting about phytomer 17 were enriched in functions associated with cell proliferation (cell cycle, cell division, DNA synthesis) and genes that regulate cell proliferation (i.e., *NGAL2, GRF, IMK*). The onset of the second increase in expression of this cohort coincides with the appearance of the second ring of NRBs on stem node 17. The tissue collected for NRB transcriptome analysis from P17-P21 included both rings of NRBs. Therefore, the increase in expression of genes involved in cell proliferation later in NRB development could be associated with the formation of the second ring of NRBs.

Microscopic analysis of NRB anatomy during development showed that nascent sorghum NRBs were initiated in the stem rind below the epidermis of the pulvinus in close association with vascular bundles as found for maize crown roots (Hochholdinger et al., 2004). Lateral roots are initiated through the priming of pericycle cells, the outermost cell layer of the root stele (Torres-Martinez et al., 2019; Zhang et al., 2021; Hochholdinger et al., 2004). In contrast, shoot-borne roots in tomato initiate from differentiated primary phloem parenchyma cells that are expressing *WOX4*, a gene expressed in tomato vasculature (Omary et al., 2022). *WOX4* promotes procambial development (Ji et al., 2010), vascular cambium maintenance in Populus (Kucukoglu et al., 2017), periderm formation (Xiao et al., 2020), adventitious root development (Wang et al., 2020), and rice tiller axillary meristem maintenance (Tanaka et al., 2015). WOX4 regulates the expression of *ALTERED PHLOEM1* (Ji et al., 2010) and cytokinin signaling (Ohmori et al., 2013). In the sorghum stem, *WOX4* may contribute to NRB initiation by maintaining cells in the stem pulvinus vasculature in a meristem competent state. Gene regulatory network analysis identified a sub-module expressed early in NRB development that includes PFA5, WOX4, and SPL9. The analysis predicted connections from these TFs to genes involved in CK-signaling (*MYB3R-1, WEE1*), GA-signaling (*SbGRF15, ORE15, LUG*), and IAA-signaling (*BDL.IAA12*) and to regulators of cell proliferation (*SPL9, PDF2*, *ANT, REM, MYB3R-1*). GRFs interact with and activate ANGUSTAFOLIA (AN3/GIF1), a master regulator of leaf, root and stem cell proliferation and meristem activity (Ercoli et al., 2018; Nelissen et al., 2015; Vercruyssen et al., 2014). Expression of *SbAN3/GIF1* in NRBs suggests that activation of *GRF1* expression could in turn increase cell proliferation via interaction with *SbAN3/GIF1*. The SbAN3/GIF1:GRF1 complex was previously predicted to bind to the *ARF3* and *ARF5* promoters linking GA-signaling and IAA-signaling in the intercalary meristem (Yu et al., 2022). A similar integration of these signaling pathways could also regulate cell proliferation during NRB initiation. However, while there are similarities between the NRB GRN and intercalary meristem GRN (Yu et al., 2022), the sorghum homologs of *PFA5*, *WOX4*, *HAT14*, *ANT2* and *BDL.IAA12* were unique to the NRB GRN module.

BDL.IAA12 is known to interact with and repress ARF5.MP, a TF involved in embryonic root initiation that is activated by increased level of auxin (Schlereth et al., 2010). Localized increases in auxin required for pericycle founder cell recruitment can be abolished by inhibiting polar auxin transport (Torres-Martinez et al., 2019). The differential expression of genes encoding the auxin transporters ABCB19, PIN4 and LAX2 early in sorghum NRB development indicates that these transporters could contribute to auxin stimulated NRB initiation. Localized induction of *ABCB19*, a non-polar auxin export transporter, enhances adventitious root formation in Arabidopsis ( (Sukumar et al., 2013)). The polar auxin transporter PIN4 is expressed in root meristems (Friml et al., 2002)) which may help shape auxin maxima at sites of NRB initiation, while the auxin importer LAX2 could increase auxin levels in or near nodal root bud founder cells. Taken together, increases in IAA mediated by LAX2/PIN4 could induce ARF activity stimulating NRB initiation as previously proposed in other plant species.

In rice, WOX11 is an important regulator of lateral/crown/adventitious root formation (Zhao et al., 2009; Liu et al., 2014a; Hu and Xu, 2016; Zhou et al., 2017; Baesso et al., 2018; Zhang et al., 2018). *WOX11* expression is induced by auxin (Zhang et al., 2018); (Zhang et al., 2018) and mutation of *WOX11* in rice reduced the number of crown roots formed and the growth rate of crown roots following emergence (Zhao et al., 2009; Qian et al., 2019). WOX11 activates expression of *LBD16* and *LBD29*, key regulators of lateral and crown root formation (Liu et al., 2014b), and *WOX5*, a *WUS*-gene involved in repression of cell differentiation in the crown root quiescent center (QC) (Zhao et al., 2017). Expression of sorghum homologs of *WOX11* and *WOX5* increased early (by P9) in NRB development to a plateau that was maintained throughout NRB development (**Figure 5**). SbWOX11 could play a similar role in sorghum NRB formation by activating expression of LBD-genes such as *LBD29*, a gene required for crown root formation in rice (Inukai et al., 2005) and maize (Taramino et al., 2007).

*PLETHORA* genes (*PLT3*, *PLT5*, *PLT7*) function early in lateral root formation (Hofhuis et al., 2013) to regulate asymmetric periclinal cell division in incipient root primordia (Du and Scheres, 2017) and lateral root spacing (Hofhuis et al., 2013). PLT3/5/7 are also required for activation of *PLT1*/*2*/*4* later in lateral root development that contributes to root meristem stem cell maintenance (Du and Scheres, 2017). In sorghum NRBs, two homologs of *PLT5* were expressed at higher levels than *PLT2* and *PLT4* early in development although all four PLTs show a pattern of increasing expression during NRB development (**Figure 5**).

Sorghum homologs of *LBD12*, *17*, and *29* were expressed at low levels early in NRB development followed by an increase in expression from node 10 to 21 (**Figure 5**). *SbLBD29* was expressed at the high levels relative to *LBD12* and *LBD17*, consistent with its central role in crown root formation (Inukai et al., 2005; Taramino et al., 2007). The maize *LBD29* homolog *RTCS* is induced by auxin signaling through ARF34, a homolog of Arabidopsis *ARF7* (Xu et al., 2015). *SbARF10* may serve this role during NRB development in sorghum. *SbLBD29* expression increases ~30-fold during sorghum NRB development in parallel with the increasing size and development of NRBs. LBD29 was found to repress the expression of NAC master regulators of secondary cell wall formation on fiber cells (Lee et al., 2019) consistent with low expression of genes involved in secondary cell wall formation during sorghum NRB formation. SbLBD29 binding sites were identified in the promoter of *NST1*, a NAC factor regulator of secondary cell wall formation indicating LBD29 could contribute to repression of secondary cell wall formation during NRB development.

ROOT MERISTEM GROWTH FACTORs (RGFs) are peptide hormones that play key roles in root apical meristem development and maintenance of the root stem cell niche (Matsuzaki et al., 2010; Ou et al., 2016). RGF action is mediated through interaction with LRR receptor-like kinases (RGIs) (Ou et al., 2022). RGF-RGI signaling regulates root meristem development in part by inducing *PLT1* and *PLT2* expression (Ou et al., 2022). Sorghum homologs of genes encoding RGFs and RGI receptors showed increasing expression during NRB development, indicating this auxin independent signaling system plays an important role in NRB-ANR development (**Figure 5**).

The long duration of sorghum NRB development prior to nodal root outgrowth indicates that NRB outgrowth is a highly regulated event. Potential repressors of outgrowth included GA2 oxidases that could maintain low levels of GA in NRB tissues. Several genes involved in strigolactone biosynthesis (*MAX1, MAX4*) showed increased expression late in NRB development, indicating strigolactones could modulate NRB outgrowth (Al-Babili and Bouwmeester, 2015; Arite et al., 2012; Brewer et al., 2016). Several genes involved in ABA-signaling show elevated levels of expression during NRB development. For example, TINY, an ABA-regulated AP2-domain TF that represses growth (Wei et al., 2005) and several genes that are involved in ABA-signaling (*EEL, ATHB17, ATHB21, ATHB40*) were expressed during NRB development. ATHB17 is a positive regulator of ABA signaling (Park et al., 2013) and ATHB21 and ATHB40 were previously associated with repression of axillary bud outgrowth (Gonzalez-Grandio et al., 2017). Expression of *AP2* and *FUL*, genes involved in meristem arrest (Martinez-Fernandez et al., 2020; Wang et al., 2023; Merelo et al., 2022) in NRBs could indicate these TFs down-regulate meristem activity.

### Study limitations

The current study of NRB development began when white dots corresponding to nascent NRBs were first visible on the pulvinus of the stem node of the 7^th^ phytomer. Therefore, earlier developmental events that specify the location of NRB initiation and the dynamics of the formation of the ring of NRBs around the circumference of the stem relative to the tiller bud were not examined in the current study. In addition, development of the second ring of NRBs starting on the 17^th^ stem node requires further study. Gene regulatory network analysis identified regulatory modules involved in NRB initiation and later stages of NRB development (root cap development). Information on the regulation of root niche/QC formation and LBD/PLT action would be aided by cell specific RNAseq analysis and targeted mutation/over-expression studies. Extension of the current study to the regulation of nodal root outgrowth, aerial root formation, and nodal root penetration of the soil profile/initiation of lateral root proliferation would help fill additional gaps in our understanding of bioenergy sorghum root system development.

## Data Availability

Data from this study are hosted by Dryad and can be accessed with the following link, DOI:10.5061/dryad.rbnzs7hhn. The data is also hosted by the JGI Genome Portal https://genome.jgi.doe.gov/portal. Below are the JGI project names and the JGI project ID that can be used to search for the datasets: Sorghum bicolor TX08001 Nodal Root Tissue Development Gene Expression Profiling, JGI Project ID: 1390741.

## Supplementary material

Supplementary Data Sheet 1 NRB gene regulatory network file.

Supplementary Data Sheet 2 NRB cytoscape gene regulatory network file.

Supplementary Data Sheet 3 Analysis of first tier neighbors of NRB gene regulatory network (GRN) TFs with high expression early in NRB development. TFs that are connected to and could therefore regulate TFs expressed early in NRB development are listed in files labeled ‘activators first neighbors’. Genes that are connected to that could be regulated by TFs expressed early in development are listed in files labeled ‘activated first neighbors’.

Supplementary Data Sheet 4 Analysis of first tier neighbors of NRB GRN TFs with high expression late in NRB development. TFs that are connected to and could therefore regulate TFs expressed later in NRB development are listed in files labeled ‘activators first neighbors’. Genes that are connected to that could be regulated by TFs expressed later in development are listed in files labeled ‘activated first neighbors’.
