# Supplementary figures and images for: Bioenergy sorghum nodal root bud development: morphometric, transcriptomic and gene regulatory network analysis
Source: Front Plant Sci. 2024 Oct 21;15:1456627. doi: 10.3389/fpls.2024.1456627 (PMC11532172; doi:10.3389/fpls.2024.1456627)

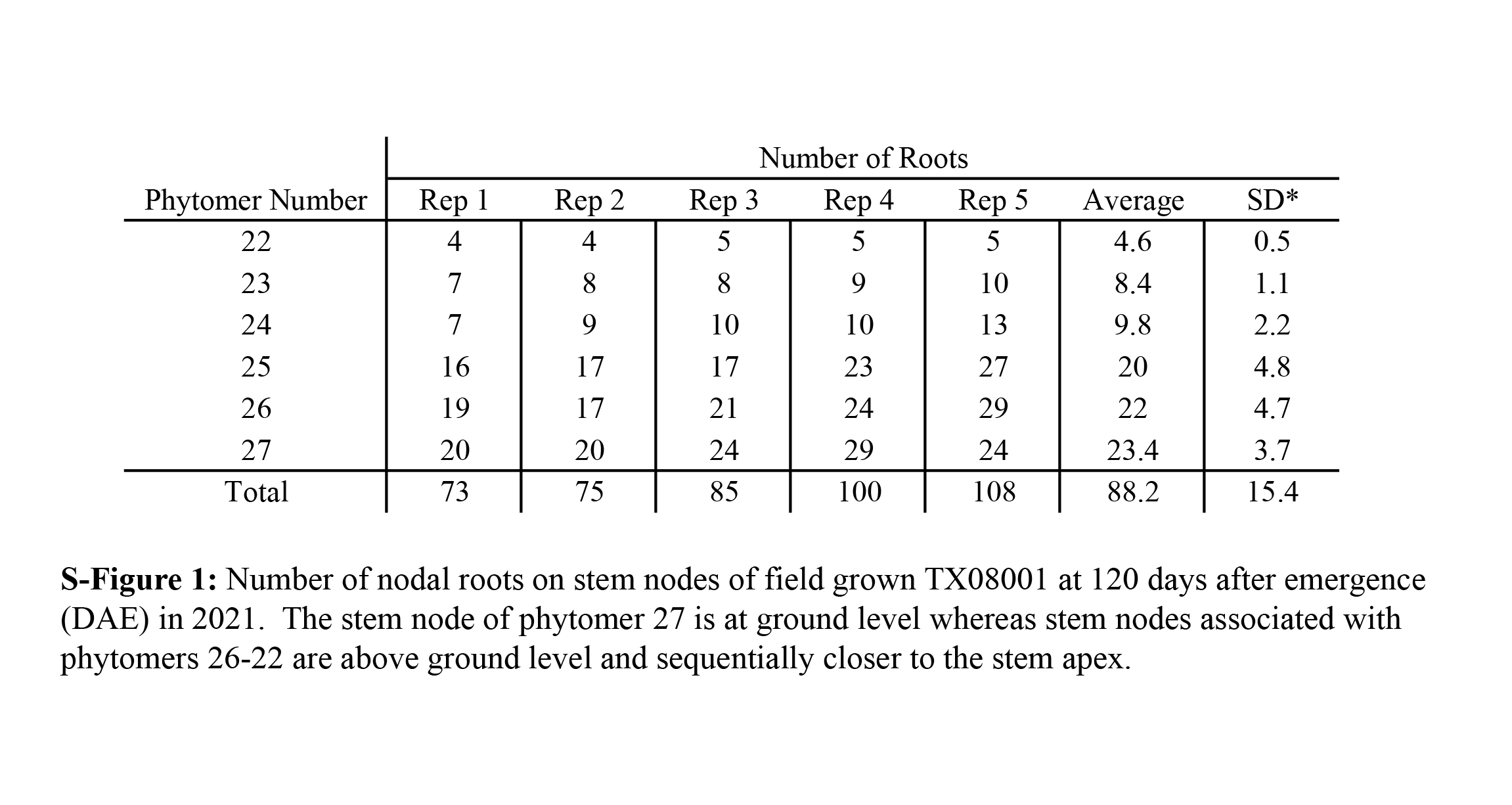

Supplement: Supplementary file 5 [file Image1.jpeg]

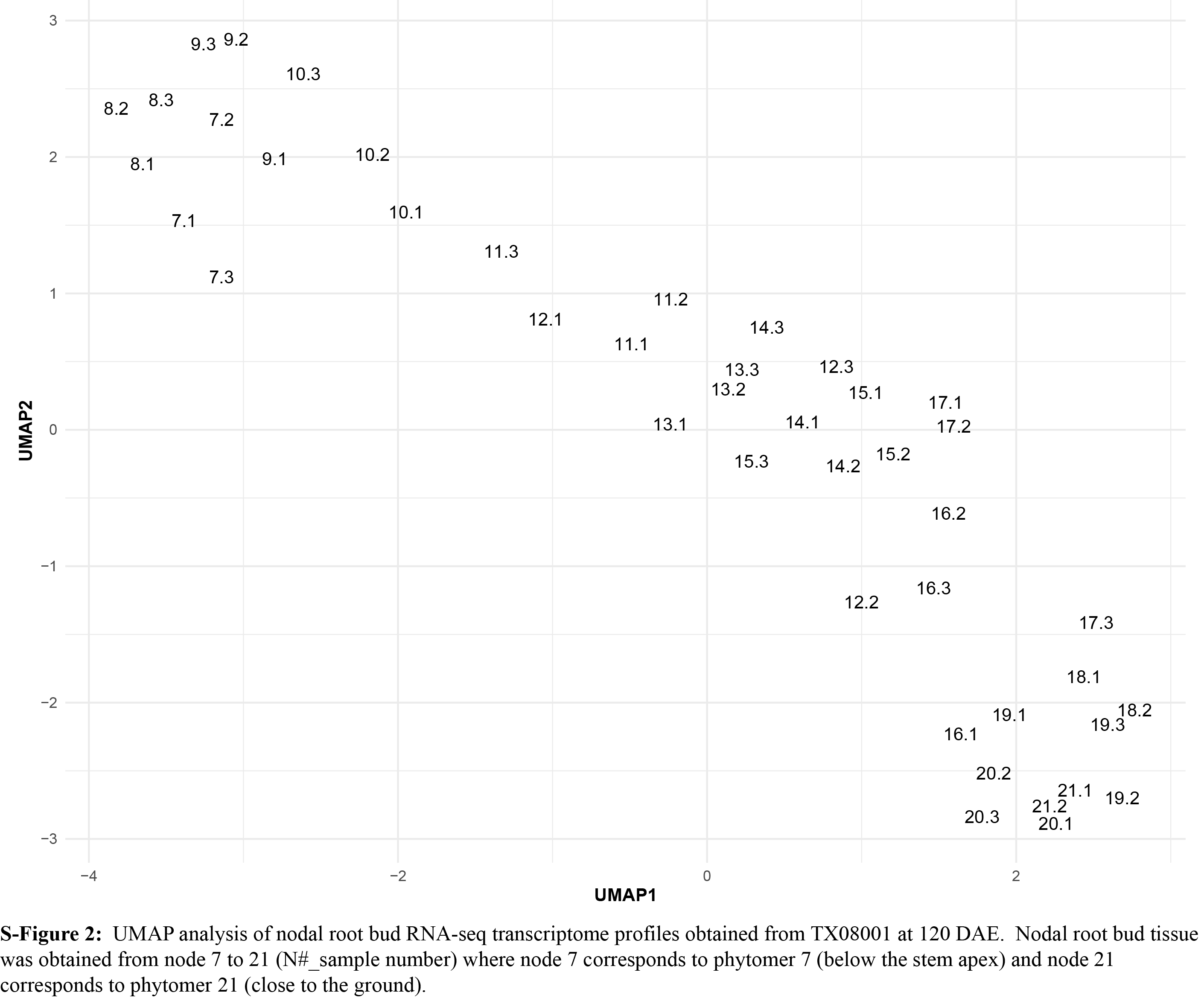

Supplement: Supplementary file 6 [file Image2.jpeg]

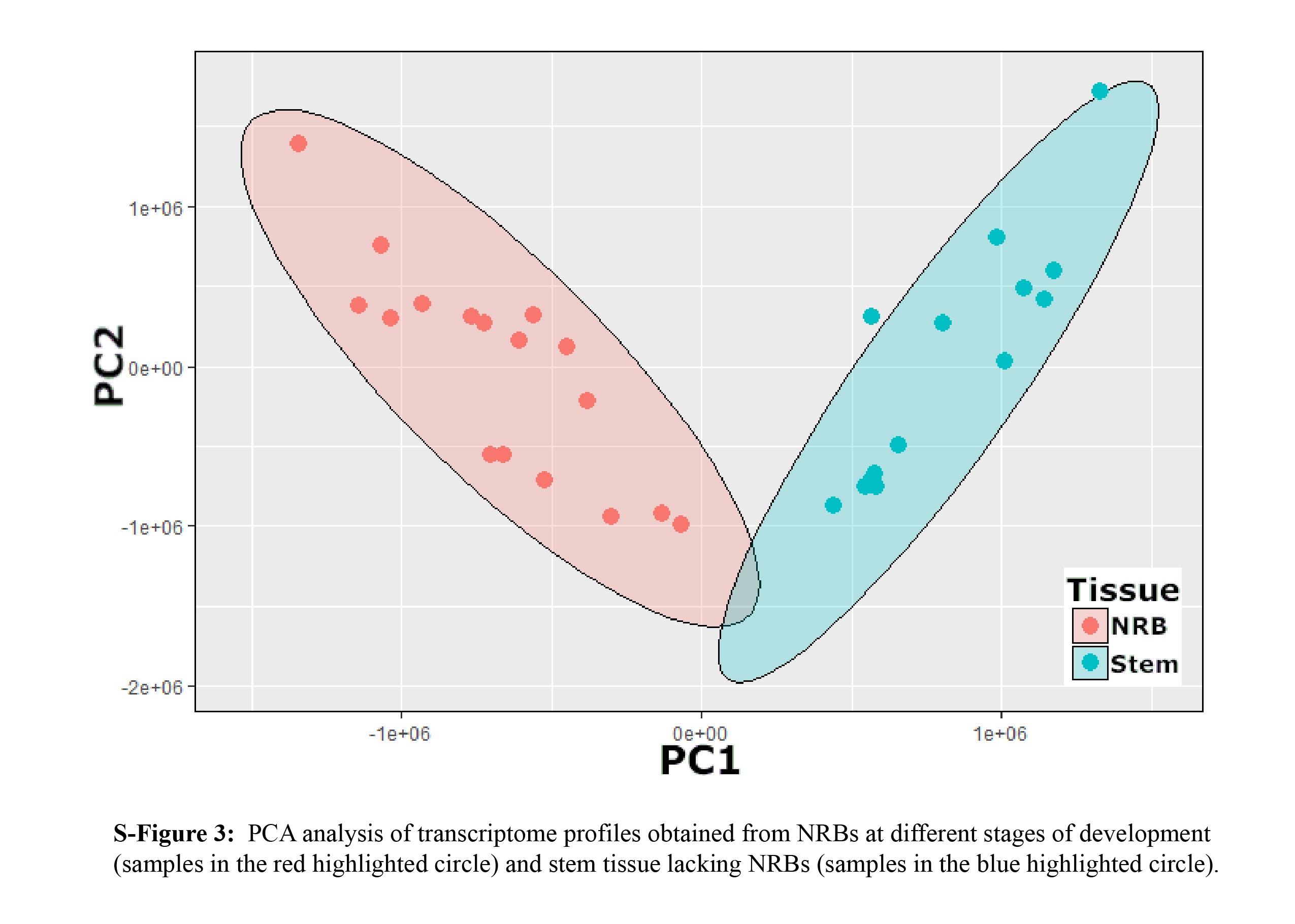

Supplement: Supplementary file 7 [file Image3.jpeg]

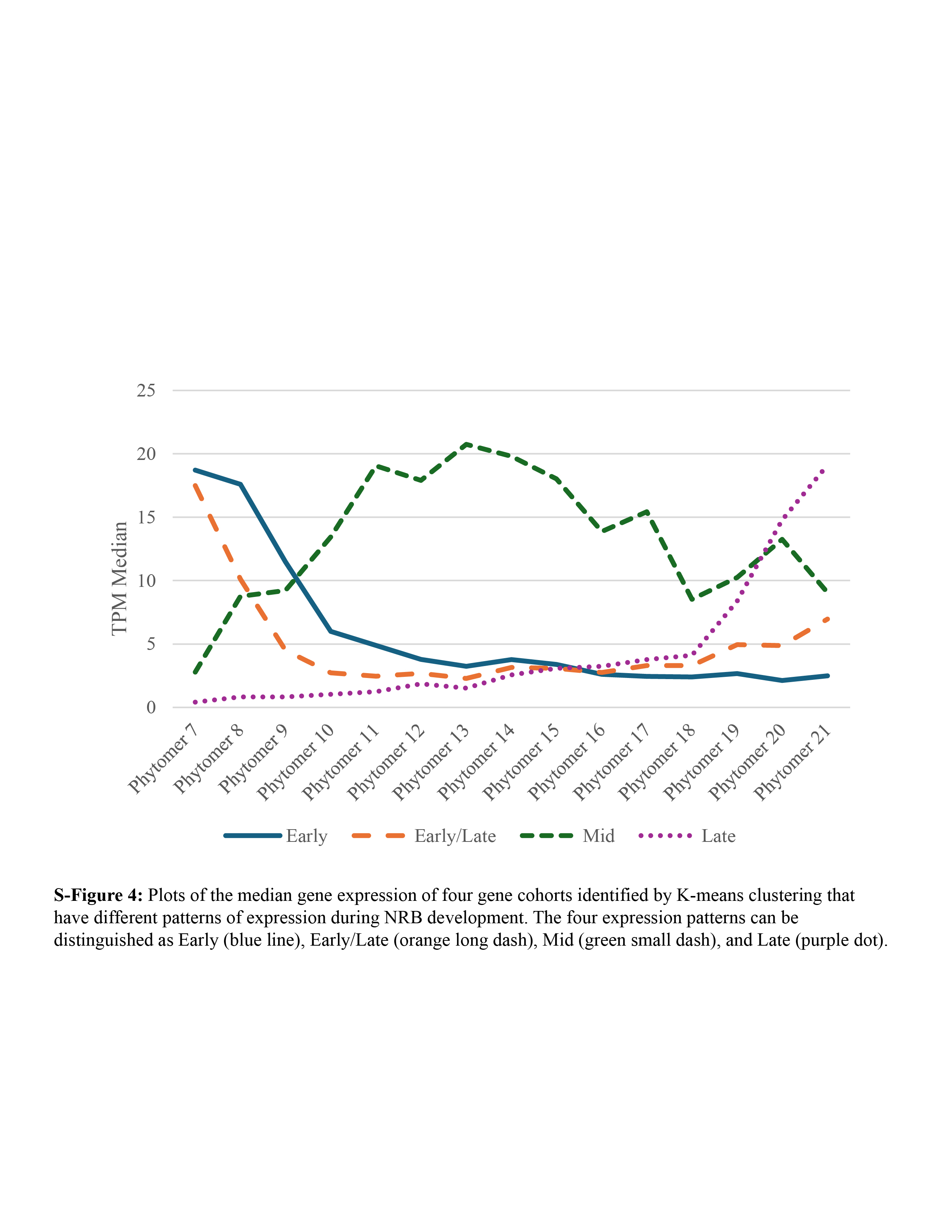

Supplement: Supplementary file 8 [file Image4.jpeg]

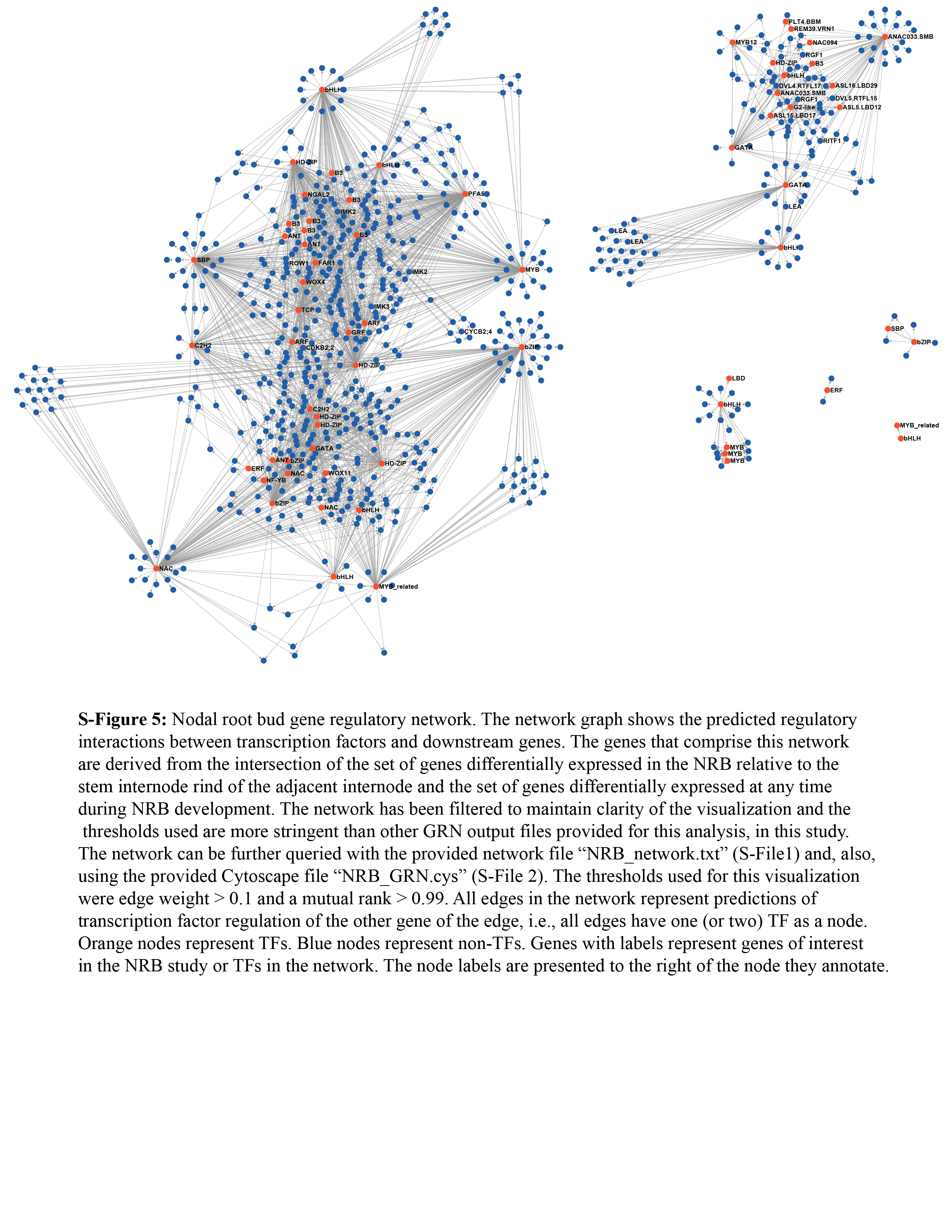

Supplement: Supplementary file 9 [file Image5.jpeg]
